# Classifications of the Dental Schools of the United States

**Published:** 1921-02

**Authors:** 


### CLASSIFICATIONS OF THE DENTAL SCHOOLS OF THE UNITED STATES

BY THE DENTAL EDUCATIONAL COUNCIL OF AMERICA

---

*Class A*

University of Southern California, College of Dentistry, Los Angeles, Cal.; University of California, College of Dentistry, San Francisco, Cal.; Northwestern University Dental School, Chicago, Ill.; University of Illinois, College of Dentistry, Chicago, Ill.; University of Iowa, College of Dentistry, Iowa City, Iowa; Harvard University Dental School, Boston, Mass.; Tufts Dental College, Boston, Mass.; University of Michigan, College of Dentistry, Ann Arbor, Mich.; University of Minnesota, College of Dentistry, Minneapolis, Minn.; St. Louis University Dental School, St. Louis, Mo.; Creighton University, College of Dentistry, Omaha, Neb.; University of Buffalo, Dental Department, Buffalo, N. Y.; Ohio State University, College of Dentistry, Columbus, Ohio; North Pacific Dental College, Portland, Ore.; University of Pittsburgh, College of Dentistry, Pittsburgh, Pa.; The Thomas W. Evans, Museum and Dental Institute, University of Pennsylvania, Philadelphia, Pa.; Vanderbilt University, School of Dentistry, Nashville, Tenn.; Medical College of

Virginia, School of Dentistry, Richmond, Va.; Marquette University, College of Dentistry, Milwaukee, Wis.

*Class B*

College of Physicians and Surgeons, Dental Department, San Francisco, Cal.; Colorado College of Dental Surgery, Denver, Col.; Georgetown University, School of Dentistry, Washington, D. C.; George Washington University Dental School, Washington, D. C.; Howard University Dental School, Washington D. C.; Atlanta-Southern Dental College, Atlanta, Ga.; University of Louisville, College of Dentistry, Louisville, Ky.; Chicago College of Dental Surgery, Chicago, Ill.; Indiana Dental College, Indianapolis, Ind.; Loyola University, School of Dentistry, New Orleans, La; Tulane University, School of Dentistry, New Orleans, La.; Baltimore College of Dental Surgery, Baltimore, Md.; University of Maryland, Dental Department, Baltimore, Md.; Washington University Dental School, St. Louis, Mo.; Kansas City-Western Dental College, Kansas City, Mo.; University of Nebraska, College of Dentistry, Lincoln, Neb.; New York College of Dentistry, New York, N. Y.; College of Dental and Oral Surgery of New York, New York, N. Y.; Western Reserve University, Dental School, Cleveland, Ohio; Ohio College of Dental Surgery, Cincinnati, Ohio; Philadelphia Dental College, Philadelphia, Pa.; University of Tennessee, College of Dentistry, Memphis, Tenn.; Meharry Dental College, Nashville, Tenn.; Baylor University, College of Dentistry, Dallas, Texas.

*Class C*

Jersey City Dental College, Jersey City, N. J.; University of West Tennessee, Memphis, Tenn.; Texas Dental College, Houston, Texas; Cincinnati College of Dental Surgery, Cincinnati, Ohio.

NOTE—These classifications were all made upon the basis of the requirements in force prior to April 30, 1920.

John V. Conzett, President; Thomas J. Barrett, Vice-

President; Albert L. Midgley, Secretary-Treasurer, 315 Butler Exchange, Providence, R. I.; Henry L. Banzhaf, Thomas A. Broadbent, John H. Baldwin, Sheppard W. Foster, H. Edmund Friesell, Clarence J. Grieves, Louis Meisburger, Arthur R. Melendy, C. A. Nixon, C. Victor Vignes, Frederick C. Waite, Herbert L. Wheeler.

